# Elevated MUC5AC expression is associated with mismatch repair deficiency and proximal tumor location but not with cancer progression in colon cancer

**DOI:** 10.1007/s00795-020-00274-2

**Published:** 2020-12-29

**Authors:** Sebastian Dwertmann Rico, Doris Höflmayer, Franziska Büscheck, David Dum, Andreas M. Luebke, Martina Kluth, Claudia Hube-Magg, Andrea Hinsch, Christina Möller-Koop, Daniel Perez, Jakob R. Izbicki, Michael Neipp, Hamid Mofid, Hannes Lárusson, Thies Daniels, Christoph Isbert, Stephan Coerper, Daniel Ditterich, Holger Rupprecht, Albert Goetz, Christoph Fraune, Katharina Möller, Anne Menz, Christian Bernreuther, Till S. Clauditz, Guido Sauter, Ria Uhlig, Waldemar Wilczak, Ronald Simon, Stefan Steurer, Patrick Lebok, Eike Burandt, Till Krech, Andreas H. Marx

**Affiliations:** 1 Institute of Pathology, University Medical Center Hamburg-Eppendorf, Martinistr. 52, 20246 Hamburg, Germany; 2 General, Visceral and Thoracic Surgery Department and Clinic, University Medical Center Hamburg-Eppendorf, Hamburg, Germany; 3 General, Vascular and Visceral Surgery Clinic, Itzehoe Medical Center, Itzehoe, Germany; 4 General, Visceral Thoracic and Vascular Surgery Clinic, Regio Clinic Pinneberg, Pinneberg, Germany; 5 General, Visceral and Tumor Surgery Clinic, Albertinen Hospital, Hamburg, Germany; 6 Department of General, Gastrointestinal and Colorectal Surgery, Amalie Sieveking Hospital, Hamburg, Germany; 7 Department of Surgery, General Hospital Martha-Maria Nuernberg, Nuernberg, Germany; 8 Department of Surgery, General Hospital Neustadt/Aisch, Neustadt an der Aisch, Germany; 9 Department of Thoracic Surgery, Academic Hospital Neumarkt, Neumarkt, Germany; 10 Department of Surgery, General Hospital Roth, Roth, Germany; 11 Institute of Pathology, Clinical Center Osnabrueck, Osnabrück, Germany; 12 Department of Pathology, Academic Hospital Fuerth, Fuerth, Germany

**Keywords:** MUC5AC, Mismatch repair deficiency, Colon cancer, Progression, Tissue microarray

## Abstract

Mucin 5AC (MUC5AC) is a secreted gel-forming mucin expressed by several epithelia. In the colon, MUC5AC is expressed in scattered normal epithelial cells but can be abundant in colorectal cancers. To clarify the relationship of MUC5AC expression with parameters of tumor aggressiveness and mismatch repair deficiency (dMMR) in colorectal cancer, a tissue microarray containing 1812 colorectal cancers was analyzed by immunohistochemistry. MUC5AC expression was found in 261 (15.7%) of 1,667 analyzable colorectal cancers. MUC5AC expression strongly depended on the tumor location and gradually decreased from proximal (27.4% of cecum cancers) to distal (10.6% of rectal cancers; *p* < 0.0001). MUC5AC expression was also strongly linked to dMMR. dMMR was found in 21.3% of 169 cancers with MUC5AC positivity but in only 4.6% of 1051 cancers without detectable MUC5AC expression (*p* < 0.0001). A multivariate analysis showed that dMMR status and tumor localization predicted MUC5AC expression independently (*p* < 0.0001 each). MUC5AC expression was unrelated to pT and pN status. This also applied to the subgroups of 1136 proficient MMR (pMMR) and of 84 dMMR cancers. The results of our study show a strong association of MUC5AC expression with proximal and dMMR colorectal cancers. However, MUC5AC expression is unrelated to colon cancer aggressiveness.

**Supplementary Information:**

The online version contains supplementary material available at 10.1007/s00795-020-00274-2.

## Introduction

Colorectal cancer was the third most common cancer worldwide in 2018 and the second most common cause for cancer related death [1]. Standard treatment of colorectal cancer consists of surgical removal. In high-risk cancers, adjuvant chemotherapy is also given in order to destroy micrometastasis and to reduce the risk of local recurrence. Possible chemotherapies include conventional cytotoxic chemotherapy and several antiangiogenic substances. In case of BRAF, KRAS, and NRAS wild-type cancers, anti-EGFR therapy antibodies can also be applied (summarized in [2]). Immune checkpoint inhibitors can be administered in cancers harboring microsatellite instability (MSI) or mismatch repair deficiency (dMMR) (summarized in [3]). Established prognostic factors of colorectal carcinomas include pT, pN, M status and histologic tumor features [4, 5]. They are statistically powerful but cannot reliably predict disease course in individual patients.

Mucin 5AC (MUC5AC) is a biomarker of potential interest in colorectal cancer. MUC5AC is a secreted gel-forming mucin [6, 7] expressed in normal mucus-producing cells of the stomach, the lung, and the uterine cervix [8–10] as well as in cancer cells of the ovarian, the pancreas, and the gastrointestinal tract [11–14]. MUC5AC expression was earlier described to occur in 0–95% of colorectal cancers in studies analyzing 22–649 cancers [11, 12, 15–35], and it was shown to be linked to MSI or dMMR in studies analyzing 35–649 cancers [16–18, 20, 22, 26, 28, 30, 33, 34]. Since MSI is linked to favorable prognosis in colorectal cancer [36], a favorable disease course could be expected in MUC5AC-positive cancers. However, multiple studies have provided evidence that MUC5AC expression may drive cancer aggressiveness in colorectal cancer cell lines and xenograft models [37–39]. The 11 studies analyzing the prognostic relevance of MUC5AC expression in colorectal cancer in cohorts of 35–649 patients have found divergent results [11, 12, 17, 18, 20, 21, 23, 25, 31, 34, 35].

None of the earlier studies on the putative clinical role of MUC5AC expression in colon cancer have considered the association between MUC5AC expression and clinic-pathological parameters in the subgroups of mismatch repair proficient (pMMR) and dMMR cancers in their analyses. We thus analyzed the relationship between MUC5AC expression and features of tumor aggressiveness (pT and pN) in all cancers and in the subgroup of pMMR and dMMR cancers in a cohort of 1812 colorectal cancers by immunohistochemistry (IHC) in a tissue microarray (TMA) format.

## Material and methods

### Tissue microarray (TMA)

Our colon cancer TMA consisted of 1,812 colon cancers diagnosed at the Institutes of Pathology of the University Medical Center Hamburg-Eppendorf (Hamburg, Germany) and the Department of Pathology of the Academic Hospital Fuerth (Fuerth, Germany) between 2009 and 2019. TMA construction was done as previously described [40]. Clinical, pathological and molecular parameters were obtained from patient records (Table 1). The use of archived remnants of diagnostic tissues for manufacturing of tissue microarrays and their analysis for research purpose as well as patient data analysis has been approved by local laws (HmbKHG, §12) and by the local ethics committee (Ethics Commission Hamburg, WF-049/09). All work has been carried out in compliance with the Helsinki Declaration.

**Table 1:** Patient cohort

	All tumors (*n* = 1812)
Age	
Median	73.2
Mean	72.1
Tumor localization	
Caecum	172 (9.6%)
* c. ascendens*	200 (11.2%)
* c. transversum*	110 (6.2%)
* c. descendens*	115 (6.5%)
* c. sigmoideum*	725 (40.7%)
Rectum	461 (25.9%)
Colon side	
Left	1311 (73.1%)
Right	483 (26.9%)
Tumor stage	
pT1	76 (4.3%)
pT2	354 (19.8%)
pT3	989 (55.4%)
pT4	365 (20.5%)
Lymph node status	
pN−	926 (52.4%)
pN +	841 (47.6%)
Mismatch repair status	
Deficient	94 (7.2%)
Proficient	1203 (92.3%)

*c.* colon

### Immunohistochemistry (IHC)

Freshly prepared TMA sections were immunostained on one day in one experiment. Slides were deparaffinized and exposed to heat-induced antigen retrieval for 5 min in an autoclave at 121 °C in pH 7.8 Dako Target Retrieval Solution buffer (Dako, Glostrup, Denmark). Primary antibody specific against MUC5AC protein (mouse monoclonal, MSVA-109, MS Validated Antibodies, Hamburg, Germany) was applied at 37 °C for 60 min at a dilution of 1:200. Bound antibody was then visualized using the EnVision Kit (Dako, Glostrup, Denmark) according to the manufacturer’s instructions. For immunostaining of the MMR proteins, primary antibodies (ready to use) specific for MLH1 (clone ES05, mouse), PMS2 (clone EP51, rabbit), MSH2 (clone FE11, mouse), and MSH6 (clone EP49, rabbit) from Dako (Glostrup, Denmark) were applied for 20 min (MLH1, MSH2, MSH) or 30 min (PMS2) in an automated immunostainer (Dako/Agilent Autostainer Link 48 (Santa Clara, USA). MUC5AC staining was seen in the membrane and cytoplasm of the colon cancer cells and immunostaining was interpreted as follows: Negative: no staining at all tumor cells, weak staining: staining intensity of 1 + in ≤ 70% of the tumor cells or staining intensity of 2 + in ≤ 30% of the tumor cells, moderate staining: staining intensity of 1 + in > 70% of the tumor cells, staining intensity of 2 + in > 30% but in ≤ 70% of the tumor cells or staining intensity of 3 + in ≤ 30% of the tumor cells, strong staining: staining intensity of 2 + in > 70% of the tumor cells or staining intensity of 3 + in > 30% of the tumor cells. Nuclear staining of the MMR proteins was interpreted as negative (no staining) and positive (at least weak staining).

### Statistics

Statistical calculations were performed with JMP^®^ software (SAS Institute Inc., NC, USA). Contingency tables and the Chi-square test were performed to search for associations between MUC5AC expression, clinical-pathological parameters, and MSI. Multinominal logistic regression was performed to test the impact of MUC5AC and tumor localization (right/left, proximal to distal) on dMMR status and test the impact of dMMR and tumor localization (right/left, proximal to distal) on MUC5AC status. A *p *value ≤ 0.05 was regarded as statistically significant.

## Results

### Technical issues

MUC5AC expression was informative in 1667 (92%) of the 1,812 arrayed cancers in our IHC analysis. Reasons for non-informative cases (*n* = 145; 8%) included lack of tissue samples or the absence of unequivocal cancer cells in the TMA spot.

### MUC5AC expression and tumor phenotype

**Fig. 1:**
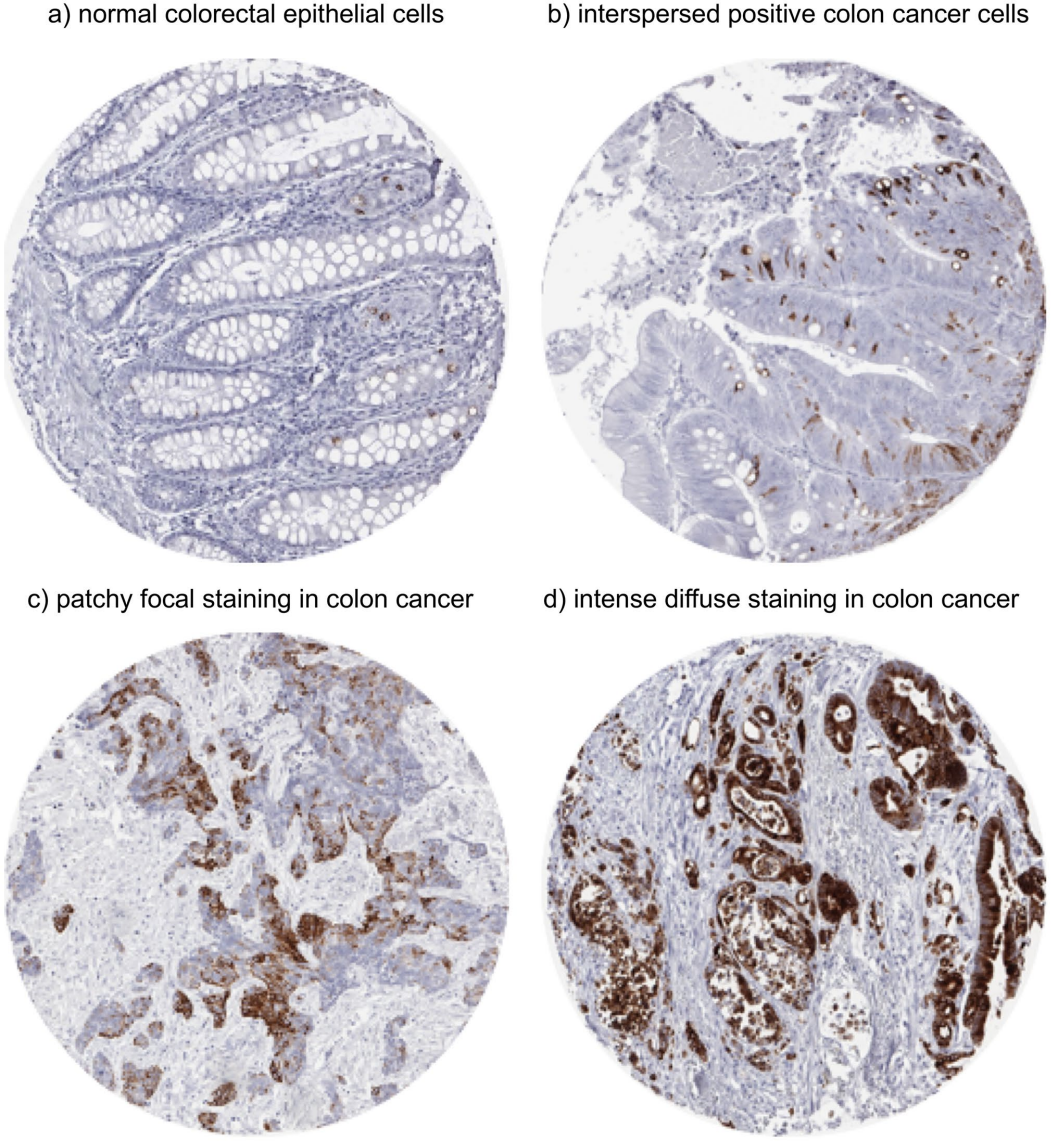
MUC5AC staining in normal and cancerous tissue (magnification: 10×)

**Fig. 2:**
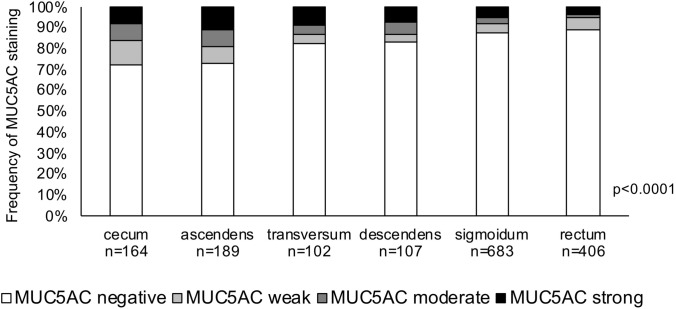
MUC5AC expression and colorectal cancer localization

In normal colorectal epithelial cells, MUC5AC expression was only seen in few scattered epithelial cells (Fig. 1a). In colorectal cancer, positive MUC5AC staining was seen in 261 (15.7%) of 1667 analyzable tumor spots. The staining patterns included variable numbers of interspersed positive cells, patchy focal staining, and intense diffuse positivity (Fig. 1b–d). According to our classification, positive cases included 97 (5.8%) cancers with weak, 63 (3.8%) with moderate, and 101 (6.0%) with strong MUC5AC staining. MUC5AC positivity was significantly associated with colorectal cancer localization. The positivity rate gradually decreased from proximal (27.4% of 164 cecum cancers) to distal (10.6% of 406 rectal cancers; *p* < 0.0001; Fig. 2). MUC5AC expression was unrelated to pT and pN (Table 2).

**Table 2:** MUC5AC expression and tumor phenotype

	*N*	Negative (%)	Weak (%)	Moderate (%)	Strong (%)	*p*	
MUC5AC staining in all cancers							
Tumor stage						0.3387	
pT1	70	87.1	8.6	1.4	2.9		
pT2	336	83.6	5.1	5.1	6.3		
pT3	907	85.7	5.8	3.0	5.5		
pT4	338	81.7	5.9	5.0	7.4		
Lymph node status						0.4523	
pN−	852	84.0	5.5	3.6	6.8		
pN +	782	85.0	6.0	4.0	5.0		
Side						< 0.0001	
Left	1204	88.0	4.7	2.5	4.8		
Right	457	74.8	8.8	7.2	9.2		
Mismatch repair status						< 0.0001	
Deficient	84	57.1	7.1	9.5	26.2		
Proficient	1136	88.3	5.7	2.5	3.5		
MUC5AC in mismatch repair deficient cancers							
Tumor stage						0.0793	
pT1	6	83.3	0	0	16.7		
pT2	19	36.8	10.5	26.3	26.3		
pT3	40	52.5	7.5	7.5	32.5		
pT4	19	78.9	5.3	0	15.8		
Lymph node status						0.0581	
pN−	55	52.7	5.4	14.5	27.7		
pN +	27	63.0	11.1	0	25.9		
MUC5AC in mismatch repair proficient cancers							
Tumor stage						0.0688	
pT1	45	88.9	11.1	0	0		
pT2	240	91.3	5.0	2.5	1.3		
pT3	618	88.7	5.5	2.1	3.7		
pT4	222	85.1	5.8	3.6	5.4		
Lymph node status						0.7912	
pN−	574	89.4	5.2	2.1	3.3		
pN +	541	87.6	6.1	2.7	3.5		

### MUC5AC expression and mismatch repair deficiency (dMMR)

dMMR as a surrogate for MSI was detected by MLH1, MSH2, PMS2, and MSH6 IHC. In brief, MLH1 was negative in 12.3% and positive in 87.7% of 1379 analyzable cases, MSH2 was negative in 2.3% and positive in 97.7% of 1356 analyzable cases, MSH6 was negative in 3.9% and positive in 96.1% of 1416 analyzable cases, and PMS2 was negative in 9.9% and positive in 90.1% of 1401 analyzable cases (Supplementary Table 1 and supplementary Fig. 1). Overall, dMMR was found in 94 (7.2%) and pMMR in 1203 (92.8%) of 1297 cases analyzable for all four proteins. Of these cases, 1220 cancers were analyzable for both MMR and MUC5AC. MUC5AC expression was significantly associated with dMMR. dMMR was found in 21.3% of 169 cancers with MUC5AC positivity but in only 4.6% of 1051 cancers without detectable MUC5AC expression (*p* < 0.0001, Table 2). This association was independent of the tumor localization, as the frequency of dMMR cancers is higher in proximal and distal colorectal tumors with MUC5AC positive in comparison with MUC5AC-negative cancers. Lack of significance in some subgroups may be due to low case numbers (Table 3). In addition, our multivariate analyses showed that firstly MUC5AC expression and tumor localization independently predicted dMMR status and secondly dMMR status and tumor localization independently predicted MUC5AC expression status (Table 4). In the subgroups of dMMR and pMMR cancers, MUC5AC expression was unrelated to pT and pN status (Table 2).

**Table 3:** MUC5AC expression, tumor localization, and mismatch repair status

MUC5AC	*N*	Mismatch repair status		*P*
		Deficient (%)	Proficient (%)	
Caecum				0.0004
Negative	74	8.1	91.9	
Weak	11	36.4	63.6	
Moderate	9	22.2	77.8	
Strong	9	66.7	33.3	
*C. ascendens*				< 0.0001
Negative	98	9.2	90.8	
Weak	13	15.4	84.6	
Moderate	8	62.5	37.5	
Strong	11	63.4	36.4	
*C. transversum*				0.0004
Negative	56	1.8	98.2	
Weak	3	0	100	
Moderate	3	33.3	66.7	
Strong	5	40.0	60.0	
*C. descendens*				0.7063
Negative	65	15.4	84.6	
Weak	3	0	100	
Moderate	1	0	100	
Strong	5	20.0	80.0	
*C. sigmoideum*				0.0064
Negative	453	3.5	96.5	
Weak	22	0	100	
Moderate	12	0	100	
Strong	22	22.7	77.3	
Rectum				0.4502
Negative	297	2.0	98.0	
Weak	18	0	100	
Moderate	2	0	100	
Strong	9	11.1	88.9	

*c.* colon

**Table 4:** Multivariate analysis

Parameter vs mismatch repair status (MMR)	Chi-square	*P*	Parameter vs MUC5AC	Chi-square	*p*
Model 1					
MUC5AC (negative, weak, moderate, strong)	47.8	< 0.0001	MMR (proficient, deficient)	47.8	< 0.0001
Localization (6 loci)	43.6	< 0.0001	Localization (6 loci)	33.1	0.0045
Model 2					
MUC5AC (negative, weak, moderate, strong)	49.6	< 0.0001	MMR (proficient, deficient)	49.6	< 0.0001
Left vs right	22.6	< 0.0001	Localization (left vs right)	24.6	< 0.0001

*MMR* mismatch repair, 6 loci: Caecum, caecum ascendens, caecum transversum, caecum descendens, caecum sigmoideum, rectum

## Discussion

MUC5AC expression was found in 16% of colorectal cancers in our study. This is in the lower range of data from previous studies. A total of 23 studies have earlier analyzed MUC5AC expression in colorectal cancer (Table 5) and described MUC5AC expression to occur in 0–95% analyzing 22–649 cancers [11, 12, 15–35]. Likely reasons for discrepant results include the use of different antibodies, IHC protocols, and cut-off levels or scores to define MUC5AC positivity as well as the composition of the tumor cohorts. Notably higher rates of MUC5AC expression were, for example, found in studies analyzing mucinous colorectal cancers only (MUC5AC expression in 23–90% of 32–194 tumors) [19 , 26, 30–32].

**Table 5:** Summary of previous MUC5AC studies

Author and year	Analyzable tumors	Antibody	Cut-off/score	Expression frequency (%)	Prognosis	Phenotype
Li et al. (2019) [12]	Meta-anaylsis	Different	Different	15–60	Favorable	No association
Mesa et al. (2020) [28]	88	292-M94	Score: percentage and intensity	37–59	–	–
Hiromoto et al. (2018) [19]	–	NCL-MUC-5AC	Score: percentage and intensity	26–53	–	–
Al-Khayal et al. (2016) [15]	22	sc-33667	Score: percentage and intensity	0	–	–
Betge et al. (2016) [17]	381	45M1	> 50% positive tumor cells	8–42	Favorable	–
Krishn et al. (2016) [24]	–	45M1	Score: percentage and intensity	38	–	–
Kesari et al. (2015) [11]	50	CLH2	> 30% positive tumor cells	25	–	No association
Kim et al. (2015) [22]	274	NCL-MUC-5AC	≥ 10% positive tumor cells	35	–	–
Raghoebir et al. (2014) [32]	32	45M1	≥ 5% tumor cells with high staining intensity	27–90	–	–
Tsai et al. (2015) [33]	123	CLH2	Score: percentage and intensity	55	–	–
Nishida et al. (2014) [29]	265	CLH2	≥ 20% positive tumor cells	17	–	–
Imai et al. (2013) [20]	250	CLH2	Score: percentage and intensity	30–95	Favorable	Inverse
Walsh et al. (2013) [34]	649	45M1	> 0% positive tumor cells	49	–	Associated
Khanh et al. (2013) [21]	206	CLH2	score: percentage and intensity	34	Favorable	No association
Matsuda et al. (2010) [27]	569	CLH2	/	15	–	–
Arai et al. (2007) [16]	35	CLH2	≥ 25% positive tumor cells	63	–	–
Park et al. (2006) [30]	194	CLH2	≥ 10% positive tumor cells	47	–	–
Losi et al. (2004) [26]	136	45M1	Percentage of positive cells	20–54	–	–
Kocer et al. (2002) [23]	41	45M1	Score: percentage and intensity	34	Favorable	Inverse
Biemer-Hüttmann et al. (2000) [18]	93	Neomarkers	Score: percentage and intensity	41	–	No association
Lennerz (2016) [25]	33	–	–	45	Unfavorable	–
Perez et al. (2008) [31]	35	CLH2	Score: percentage and intensity	23	Favorable	No association
Wang et al. (2017) [35]	139	NCL-MUC5-AC	> 20% positive tumor cells	28	Favorable	Associated

Our data demonstrate a striking link of MUC5AC expression with right colon tumor location and dMMR. Both findings are consistent with data from previous studies. A total of 13 studies have earlier analyzed the relationship of MUC5AC expression and tumor location in colon cancer, and almost all of them (11/13) have found a significant association of MUC5AC expression with proximal cancers (Supplementary Table 2). For example, MUC5AC expression was found by Betge et al. in 66% of 107 right-sided cancers and in 47% of 107 left-sided cancers [17], Nishida et al. in 27% of 89 right-sided cancers and in 12% of 27 left-sided cancers [29], Imai et al. in 63% of 114 right-sided cancers and in 31% of 121 left-sided cancers [20], Walsh et al. in 60% of 220 right-sided cancers and in 43% of 417 left-sided cancers [34], Park et al. in 53% of 76 right-sided cancers and in 17% of 118 left-sided cancers [30], and Biemer-Hüttmann et al. in 55% of 40 proximal and in 22% of 23 rectal cancers [18]. A gradual change of biomarker expression from proximal to distal colon could, for example, be explained by continuing changes in the density and the composition of the stool, and the exposure time to different carcinogenic factors during the colon passage or be related to the embryonal development of the colon (summarized in [41]). Other examples of biomarkers that were described to vary dependent on the localization within the colon, for example, include AMACR [42], p53 [30] as well as amplification of EGFR and HER2 [43].

At least 9 studies have analyzed MUC5AC expression and dMMR/MSI in colon cancer, and all of them have described significant associations (Supplementary Table 3). For example, MUC5AC expression was found by Betge et al. in 7% of 350 pMMR and in 17% of 23 dMMR cancers [17], Imai et al. in 43% of 72 pMMR and in 84% of 19 dMMR cancers [20], Arai et al. in 45% of 20 MSS and in 87% of 15 MSI cancers [16], Losi et al. in 47% of 23 pMMR and in 67% of 27 dMMR cancers [26], and Biemer-Hüttmann et al. in 28% of 47 MSS and 77% of 22 MSI tumors [18]. Given the well-known associations of MSI with right-sided colon cancer location (reviewed in [44]), it was expected that MUC5AC (as any biomarker) would be linked to both or none. However, our multivariate analysis revealed that tumor location and dMMR independently enhanced the likelihood for detectable MUC5AC expression in colorectal cancer. The underlying molecular mechanism is not known. However, one study hypothesizes an increased likelihood of MUC5AC promotor hypomethylation in MSI cancers. MUC5AC promotor hypomethylation results in MUC5AC upregulation and was shown to be associated with a “mutator” phenotype in—especially MSI—colon cancers [45].

In a thorough experimental study, Pothuraju et al. have recently shown that differential MUC5AC expression drives tumorigenesis and promotes aggressiveness of colorectal cancer cells in vitro and in vivo. The authors examined the impact of reduced (shRNA-mediated) or absent (CRISPR/Cas9-mediated) MUC5AC expression on cell proliferation, anchorage independent cell growth, cell migration, and cell invasion in two endogenous MUC5AC expressing colorectal cancer cell lines. In addition, they prepared a MUC5AC knockout xenograft model to investigate the impact of MUC5AC on tumorigenesis in vivo. Overall, the authors showed that MUC5AC expression enhanced cell growth, invasion and migration and decreased apoptosis in vitro and increased tumorigenesis in vivo [38]. However, the absence of associations between MUC5AC expression and pT as well as pN argues against a clinically relevant role of MUC5AC expression for driving aggressiveness of human colorectal cancer cells. A lack of clinical relevant prognostic impact of MUC5AC expression is in line with the conflicting results of 11 earlier studies analyzing the clinical relevance of MUC5AC expression in colorectal cancer (summarized in Table 5). Of these, seven studies with 35–381 patients described an association of high MUC5AC expression with favorable phenotype and/or prognosis [12, 17, 20, 21, 23, 31, 35], three studies with 33–250 patients reported a link between high MUC5AC expression and poor phenotype and/or prognosis [25, 34, 35], and five studies involving 35–206 patients could not find any relationship between MUC5AC expression and clinic-pathological features [11, 12, 18, 21, 31]. The fact that MUC5AC expression was also unrelated to aggressive cancer phenotype in our 1051 pMMR cancers demonstrates that adverse prognostic effects of MUC5AC are not obscured by the favorable prognostic influence of dMMR.

It is of note that MUC5AC may also represent a suitable drug target. Ensituximab (Neo-102), a novel chimeric monoclonal antibody, binds to an aberrantly glycosylated cancer-associated MUC5AC variant and is able to activate the immune system to exert a cytotoxic T-lymphocyte response [47]. In a phase I study of pancreatic cancer patients preselected for MUC5AC expression, a favorable toxicity profile was found for Ensituximab [47]. In a recent phase II study, Ensituximab resulted in stable disease in 21% of 56 patients with heavily pretreated refractory colorectal cancers and was well tolerated [48]. Given the high numbers of inflammatory cells occurring in MSI/dMMR-positive colorectal cancers, one might speculate that Ensituximab treatment might be potentially promising in these carcinomas.

In summary, the results of our study show that elevated MUC5AC expression is independently linked to proximal location and dMMR in colorectal cancers. However, both in dMMR and in pMMR cancers, MUC5AC expression is unrelated to aggressive cancer phenotype.

## Competing interest

The Institute of Pathology of the UKE receives royalties on the sales of MUC5AC clone MSVA-109 from MS Validated Antibodies GmbH (owned by a family member of GS).

## Ethical approval

The usage of archived diagnostic left-over tissues for manufacturing of tissue microarrays, their analysis for research purposes and patient data analysis has been approved by local laws (HmbKHG, §12,1) and by the local ethics committee (Ethics commission Hamburg, WF-049/09). All work has been carried out in compliance with the Helsinki Declaration.

## Supplementary Information

Below is the link to the electronic supplementary material.

Supplementary file1 Supplementary figure 1. MMR protein immunostaining in colorectal cancer. Supplementary table 1. Frequency of mismatch repair protein staining in colorectal cancer. Supplementary table 2. MUC5AC immunostaining and tumor location in previous studies. Supplementary table 3. MUC5AC immunostaining and mismatch repair deficiency/microsatellite instability in previous studies (DOCX 14617 KB)
