# Supplementary material for: Elevated MUC5AC expression is associated with mismatch repair deficiency and proximal tumor location but not with cancer progression in colon cancer
Source: Med Mol Morphol. 2020 Dec 29;54(2):156–65. doi: 10.1007/s00795-020-00274-2 (PMC8139930; doi:10.1007/s00795-020-00274-2)
Supplement: Supplementary file 1 — Supplementary file1 Supplementary figure 1. MMR protein immunostaining in colorectal cancer. Supplementary table 1. Frequency of mismatch repair protein staining in colorectal cancer. Supplementary table 2. MUC5AC immunostaining and tumor location in previous studies. Supplementary table 3. MUC5AC immunostaining and mismatch repair deficiency/microsatellite instability in previous studies (DOCX 14617 KB) [file 795_2020_274_MOESM1_ESM.docx]

Supplementary figure 1. MMR protein immunostaining in colorectal cancer

Supplementary table 1. Frequency of mismatch repair protein staining in colorectal cancer

|  |  | **immunostaining of MMR proteins** | | | |
| --- | --- | --- | --- | --- | --- |
|  |  | **negative n (%)** | **weak n (%)** | **moderate n (%)** | **strong n (%)** |
| MLH1 | 1379 | 170 (12.3) | 266 (19.3) | 193 (14.0) | 750 (54.4) |
| MSH2 | 1356 | 32 (2.3) | 142 (10.5) | 127 (9.4) | 1055 (77.8) |
| MSH6 | 1416 | 54 (3.9) | 159 (11.2) | 176 (12.4) | 1027 (72.5) |
| PMS2 | 1401 | 138 (9.9) | 282 (20.1) | 231 (16.5) | 750 (53.5) |
| Abbreviations: MMR: mismatch repair | | | |  |  |

Supplementary table 2. MUC5AC immunostaining and tumor location in previous studies

|  |  | **MUC5AC immunostaining** | | |
| --- | --- | --- | --- | --- |
| **author and year** | **analyzable tumors** | **left side** | **right side** | **association** |
| Li et al. 2019 | meta-anaylsis | / |  | right side |
| Mesa et al. 2020 | 61 | 11/30 (37%) | 18/31 (58%) | right side |
| Betge et al. 2016 | 214 | 50/107 (47%) | 71/107 (66%) | right side |
| Kesari et al. 2015 | 46 | 8/28 (29%) | 5/18 (28%) | no association |
| Nishida et al. 2014 | 116 | 3/27 (12%) | 24/89 (27%) | right side |
| Imai et al. 2013 | 235 | 38/121 (31%) | 72/114 (63%) | right side |
| Walsh et al. 2013 | 637 | 179/417 (43%) | 132/220 (60%) | right side |
| Khanh et a. 2013 | 206 | 27/92 (29%) | 42/114 (37%) | no association |
| Park et al 2006 | 194 | 20/118 (20%) | 40/76 (53%) | right side |
| Losi et al. 2004 | 76 |  |  | no association |
| Kocer et al. 2002 | 41 | / | / | right side |
| Biemer-Hüttmann et al. 2000 | 63 | 5/23 (22%) | 22/40 (55%) | right side |
| Perez et al. 2008 | 35 | 1/22 (5%) | 7/13 (54%) | right side |

Supplementary table 3. MUC5AC immunostaining and mismatch repair deficiency / microsatellite instability

|  |  | **MUC5AC immunostaining** | | |
| --- | --- | --- | --- | --- |
| **author and year** | **analyzable tumors** | **MSS/pMMR** | **MSI/dMMR** | **association** |
| Betge et al. 2016 | 373 | 25/350 (7%) | 4/23 (17%) | dMMR |
| Kim et al. 2015 | 274 | / | / | MSI-high |
| Raghoebir et al. 2014 | 32 | 44/95 (46%) | 23/28 (82%) | MSI |
| Imai et al. 2013 | 91 | 31/72 (43%) | 16/19 (84%) | dMMR |
| Walsh et al. 2013 | 637 |  |  |  |
| Arai et al. 2007 | 35 | 9/20 (45%) | 13/15 (87%) | MSI |
| Park et al 2006 | 194 |  |  | MSI-high |
| Losi et al. 2004 | 50 | 11/23 (47%) | 18/27 (67%) | dMMR |
| Biemer-Hüttmann et al. 2000 | 69 | 13/47 (28%) | 17/22 (77%) | MSI |
| Abbreviations: MMR: mismatch repair, d: deficiency, p: proficiency, MSI: microsatellite instable | | | | |
